# Investigating the Adsorption of Congo Red From Aqueous Solutions on Naturally Occurring and Acid‐Activated Libyan Bentonite

**DOI:** 10.1155/tswj/6075083

**Published:** 2026-05-18

**Authors:** Salha M. Aghila, Hana A. Jamhour, Abdulhakim A. Jangher, Basher M. Mahara, Ibrahim M. Hamammu, Daefalla M. Tawati

**Affiliations:** ^1^ Libyan Authority for Scientific Research, Tripoli, Libya; ^2^ Libyan Polymer Research Center, Tripoli, Libya; ^3^ Research and Consultancy Center, Sirte University, Sirte, Libya, su.edu.ly; ^4^ Libyan Advanced Occupational Center for Welding Technologies, Tripoli, Libya; ^5^ Department of Chemistry, Faculty of Sciences, University of Tripoli, Tripoli, Libya, uot.edu.ly; ^6^ Department of Physics, Faculty of Science, University of Benghazi, Benghazi, Libya, uob.edu.ly; ^7^ Department of Physics, Faculty of Sciences, Physics Research Group, University of Benghazi, Benghazi, Libya, uob.edu.ly

**Keywords:** activated, Libyan bentonite, adsorption, Congo red (CR) dyes, kinetic model, Libyan bentonite, pseudo-second order

## Abstract

This study investigates the potential of Libyan bentonite as an eco‐friendly and sustainable adsorbent for the removal of Congo red (CR) dye from aqueous solutions. Natural and acid‐activated bentonite samples were collected from the Umm al‐Razm region of Libya. Experimental conditions, such as initial CR concentrations (10–60 ppm), pH values (5–10), contact times (5–120 min), temperatures (25°C–55°C), and adsorbent doses (0.001–0.007 g), were applied to natural and acid‐activated bentonite samples drawn from Umm al‐Razm, Libya, using a batch adsorption approach. Acid activation significantly improved the adsorption performance compared to the natural material. The Freundlich isotherm model describes the adsorption of the CR onto both materials, according to equilibrium data. This conformance highlights the heterogeneous adsorption process involving multilayer formation. Furthermore, thermodynamic analysis definitively proved the process to be endothermic and spontaneous, confirming that increasing the temperature enhances the adsorption capacity. These results offer critical insights into the physicochemical modifications induced by acid activation and illustrate the basic concepts of the mechanism governing CR uptake. The findings offer important insights for developing targeted surface engineering strategies that effectively tackle the inherent electrostatic challenges faced in the design of adsorbent materials.

## 1. Introduction

The discharge of dye‐contaminated wastewater into the environment carries significant ecological and health problems. Dyes resist natural degradation because of their complex chemical compositions and excellent stability. Even at low concentrations, they drastically affect water color, impair photosynthetic processes, and destroy aquatic ecosystems [1]. Congo red (CR), a diazo dye used in sectors such as textiles, paper, and plastics, is a significant polluter. Its toxicity, mutagenicity, and ability to break down into carcinogenic chemicals under particular pH circumstances make it harmful to human health and the environment. It can disturb the digestive, circulatory, and cardiovascular systems, as well as hinder plant and animal growth [2–5]. Although traditional methods for treating dye‐contaminated water include chemical coagulation, advanced oxidation, and filtration, adsorption has been shown to be a simple, cost‐effective, and efficient alternative [2].

Natural clays, such as bentonite, kaolinite, and montmorillonite, are promising adsorbents due to their abundance, low cost, and high adsorption capacity [6]. Among them, bentonite, which is rich in smectite minerals like montmorillonite, is widely used in industrial applications. Its 2:1 layered structure has a net negative charge due to isomorphic replacements in its tetrahedral and octahedral layers, which is balanced by exchangeable cations inside its interlayer gaps [7, 8]. While bentonite is efficient against some contaminants, its hydrophilic nature inhibits its ability to absorb organic chemicals such as CR. Chemical modification techniques, such as acid activation, are commonly used to increase bentonite performance. Acid treatment with inorganic acids such as HCl replaces interlayer cations with hydrogen ions, hence increasing surface area and porosity [1]. This change considerably increases adsorption capacity, with studies reporting over 95% CR elimination effectiveness because of the availability of active adsorption sites [9, 10]. Libyan bentonite has demonstrated its effectiveness as an adsorbent for heavy metals and dyes. Research has shown that the adsorption of heavy metals like lead and nickel follows the Freundlich isotherm model, with optimal efficiency observed at pH 6–6.5 [11]. Studies on methylene blue dye removal revealed that natural Libyan bentonite (NLBn) adheres to the Langmuir isotherm model, whereas acid‐activated bentonite (ALBn) aligns with the Temkin model, achieving up to 99% removal [12].

This study examines the enhancement of Libyan bentonite′s adsorption capacity for CR dye via hydrochloric acid (HCl) activation. Batch adsorption experiments were conducted to assess the effects of adsorbent dosage, contact time, initial dye concentration, temperature, and pH, aiming to develop an efficient and sustainable approach for the removal of dye contaminants from wastewater.

## 2. Experimental

### 2.1. Reagents and Solutions

**Figure 1:**
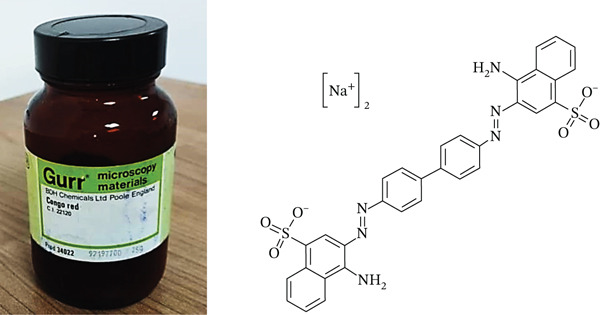
Structure of Congo red.

CR, an anionic azo dye, was used as the adsorbate in this study. The chemical formula of CR is C32H22N6Na2O6S2 with a molecular weight of 696.7 g/mol. The chemical structure of the dye is shown in Figure 1. Detailed physicochemical properties and specifications of CR are provided in [13]. The dye was used without further purification and was obtained from the Industrial Research Center (IRC), laboratory in Libya. It was originally manufactured by BDH Chemicals Ltd., Poole, England (Product Code 34022; Color Index 22120). Fourier‐transform infrared (FT‐IR) spectra were recorded using a Thermo Scientific Nicolet spectrometer.

**Figure 2:**
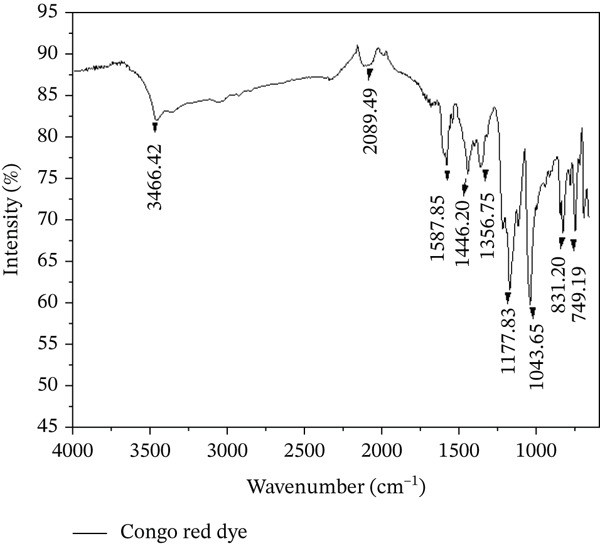
FT‐IR spectrum of CR dye.

The FT‐IR spectrum of CR (Figure 2) shows several characteristic absorption bands associated with the functional groups present in the dye molecule. A broad band observed around 3466 cm^−1^ can be attributed to O–H or N–H stretching vibrations, which may arise from moisture adsorption or intermolecular hydrogen bonding. The bands at 1587 and 1446 cm^−1^ correspond to the stretching vibrations of aromatic C=C bonds in the benzene rings of the CR structure. Additional bands in the region 1365–749 cm^−1^ are assigned to C–N and C–O stretching vibrations as well as out‐of‐plane bending vibrations of aromatic C–H groups. These spectral features are consistent with the reported FT‐IR characteristics of CR and confirm the presence of functional groups responsible for its adsorption behavior [14, 15].

### 2.2. Preparation of an Adsorbent

#### 2.2.1. Preparing the Natural Bentonite Adsorbent

Bentonite ore was extracted from the Umm al‐Razzam region of northern Libya. The raw material was crushed, ground into ≤ 50 *μ*m, and sifted as part of the preparation process. Dust and other water‐soluble contaminants were removed from the bentonite by washing it with distilled water. After cleaning, it was dried using an oven and kept for later use as a natural clay [16, 17].

#### 2.2.2. Preparing the ALBn Adsorbent

One hundred grams of natural bentonite, which had been cleaned and dried in Section 2.2.1 for preparation, was placed in a flask with 500 mL of HCl (5 M) solution. The resulting suspension was stirred constantly for 2 h at room temperature. It was soaked for 24 h until the experiment was complete, then acid and bentonite were separated by pouring the resultant slurry into a Buchner funnel. The residual bentonite was washed several times with distilled water until excess Cl^−^ ions were removed and the pH was adjusted to 6. The activated bentonite sample was dried at 105°C for 4 h. The activated samples were placed in tightly sealed plastic vials for storage until use. The alteration of XRD and FT‐IR gauges was used to monitor the bentonite structure during the acid‐activation method [18–20].

#### 2.2.3. Adsorbent Characterization

The composition of the native and ALBn samples was determined using a variety of techniques, including XRF, XRD, FT‐IR, and SEM. XRF analysis was performed using the portable Spectro X SORT XHHO3 equipment, whereas XRD analysis was performed using the Philips PW1800 model. FT‐IR analysis was performed in the 400–4000 cm^−1^ range to detect functional groups in the samples. The results of these studies, including percentages of elements, SD, and RSD, which compared the NLBn and ALBn samples, are presented in Section 3.3. Additionally, the findings from previous studies were reviewed, and SEM exploration was achieved using a JEOL JSM‐5610LV microscope from Japan.

### 2.3. Studies on Batch Adsorption

CR was adsorbed on NLBn and ALBn using various adsorption conditions, such as contact duration, adsorbent dosage, initial CR level, pH, and temperature. The characteristic absorbance of each solution was measured using a HACH DR/2400 (USA) single‐beam UV‐VIS spectrophotometer, and the concentration range under investigation for CR dye was created by serial dilutions from the standard stock solution (1000 mg/L). The maximum absorbance (*λ* max) for CR dye was determined to be 491 nm, and then a calibration curve was used to transform the absorbance into concentration. The study investigated the effect of NLBn and ALBn on CR sorption by adding 0.005 g of adsorbents to 40 mL of CR solution (initial conc. 50 mg/L, pH = 6.9) and shaking by HAAKE (003‐3678, Germany) at a rotation speed of 120 rpm. Temperature investigations were carried out at 25°C, 35°C, 45°C, and 50°C to determine the influence of adsorption. The influences of initial pH were investigated using the values 5, 6, 7, 8, 9, and 10. The dye solution pH was measured using a Jenway 3030 pH meter and adjusted using 0.1 M NaOH and HCl solutions. The adsorbent dosage employed in this investigation varied from 0.001 to 0.007 g. After equilibration, the material was separated using centrifugation (speed 6500 rpm; 10 min), and the residual CR concentration was measured using a UV spectrophotometer. Equations (1) and (2) were used to calculate the adsorbed *q*_*e*_ (mg/g) and removal proportion *R* *%* of CR:

(1) qe=Co−Cem

(2) R%=Co−CeCo×100.

Here, *C*_o_ and *C*_*e*_ represent the initial concentrations of CR in mg/L, with *C*_*e*_ also indicating the remaining concentration of the CR after the adsorption process at any given time (mg/L).

*V* denotes the volume of the CR solutions in liters (L), whereas *m* indicates the weight of the adsorbent in grams [10].

## 3. Results and Discussion

### 3.1. XRF Analysis

Tables 1 and 2 show the chemical compositions of both natural and activated bentonite samples that underwent XRF analysis. It suggests the existence of silica, alumina, calcium oxide, iron oxide, and magnesium oxide as main ingredients. Other examinations indicate the existence of residues of sodium, potassium, and titanium oxides as contaminants [16, 17].

**Table 1:** Chemical components of Libyan natural bentonite.

**Wt.%**	**SiO_2_**	**Al_2_O_3_**	**Fe_2_O_3_**	**CaO**	**V_2_O_5_**	**CuO**	**K_2_O**	**TiO_2_**	**ZnO**	**MnO**	**PbO**	**Nb_2_O_5_**	**ZrO_2_**	**SrO**
(NLBn)	66.678	19.003	10.178	0.353	0.014	0.005	2.026	1.392	0.022	0.008	0.034	0.034	0.005	0.013
RSD	63.40	38.36	83.10	67.60	1.39	100	52.70	17.28	100	77.30	80.40	43.06	9.21	30.37
SD	64.90	11.15	12.95	0.37	17.28	0.026	1.64	0.37	0.034	0.01	0.00	0.00	0.01	0.01
**Wt.%**	**Rb_2_O**	**MoO_2_**	**SO_3_**	**NiO**	**CL**	**Se**	**Br**	**Ag**	**Ti**	**Bi**	**Th**	**U**	**Y**	
(NLBn)	0.006	0.001	0.042	0.017	0.177	0.001	0.003	0.004	0.000	0.001	0.003	0.003	0.013	
RSD	13.98	24.39	100	52.70	56.80	100	46.04	72.30	100	100	96.60	100	30.37	
SD	0.001	0.001	0.065	1.640	0.160	0.026	0.002	0.004	0.001	0.001	0.005	0.004	0.006	

Abbreviations: RSD, relative standard deviation; SD, standard deviation.

**Table 2:** Chemical components of Libyan‐activated bentonite.

**Wt.%**	**SiO_2_**	**Al_2_O_3_**	**Fe_2_O_3_**	**CaO**	**V_2_O_5_**	**Cuo**	**K_2_O**	**TiO_2_**	**ZnO**	**BaO**	**PbO**	**Nb_2_O_5_**
(ALBn)	76.785	15.323	5.458	0.022	0.009	0.003	1.360	1.320	0.011	0.005	0.003	0.007
RSD	18.63	27.68	31.49	107	90.10	63.82	24.51	34.80	37.21	72.20	35.18	9.64
SD	5.84	43.32	3.51	0.048	0.02	0.00	0.57	0.84	0.01	0.01	0.00	0.00
**Wt.%**	**Rb_2_O**	**MoO_2_**	**Se**	**Br**	**Ag**	**Ti**	**Bi**	**Th**	**U**	**Y**	**—**	**—**
(ALBn)	0.004	0.001	0.001	0.001	0.004	0.000	0.000	0.002	0.003	0.002	—	—
RSD	13.98	24.39	38.23	47.94	49.39	100	100	25.06	52.7	22.81	—	—
SD	0.001	0.001	0.001	0.001	0.004	0.001	0.001	0.001	0.003	0.001	—	—

Abbreviations: RSD, relative standard deviation; SD, standard deviation.

SiO_2_ content increased from 66.68% to 76.7% in activated bentonite (ALBn) in comparison to natural (NLBn), whereas Al_2_O_3_ content decreased from 19.003% to 15.323%, and SO_3_, Cl, and Fe_2_O_3_ content significantly decreased from 0.1777 to 0.04, and from 10.178% to 5.458%, respectively, indicating the established solubility in an acidic mixture, which led to improved porous structures that enhanced clay adsorption capacity. Thus, the impact of 5% HCl activation on Umm Al‐Razam bentonite closely matches the predicted results recorded in the literature, demonstrating that the method successfully improves the bentonite quality for industrial purposes [18].

#### 3.1.1. Examination of the Produced Adsorbents Using X‐Ray Diffraction (XRD)

**Figure 3:**
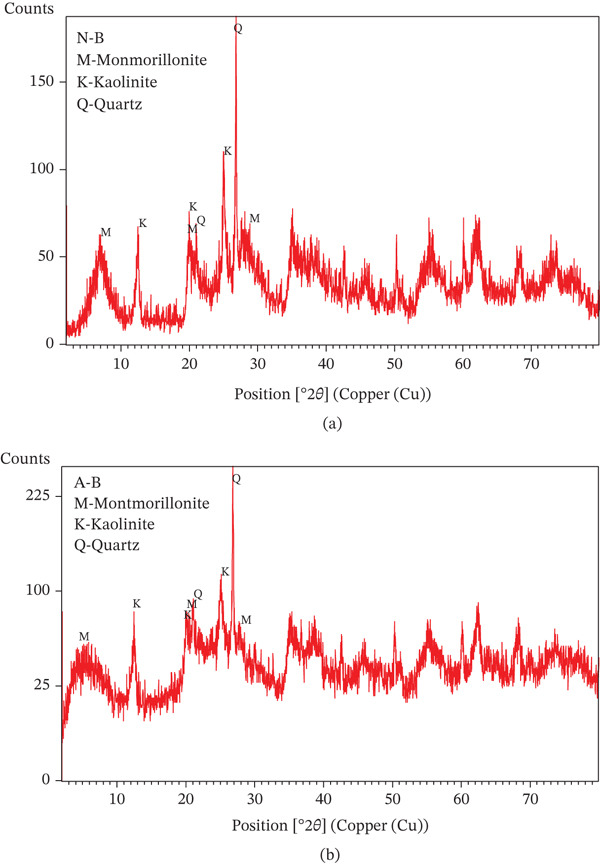
XRD spectra of (a) natural bentonite (NLBn) and (b) activated bentonite (ALBn).

The bentonite is montmorillonite, as shown by the SiO2/Al2O3 ratio of 2.71. The XRD study confirms this as well. Quartz and trace amounts of feldspar, dolomite, and calcite were the mineral phases found in addition to montmorillonite. Figure 3 displays XRD patterns of clay along with acid‐activated clay. The bulk XRD analysis of Libyan natural bentonite found that Ca‐montmorillonite was most prevalent, followed by kaolinite, with trace levels of impurities including feldspar and quartz.

#### 3.1.2. FT‐IR Analysis of the Prepared Adsorbents

**Figure 4:**
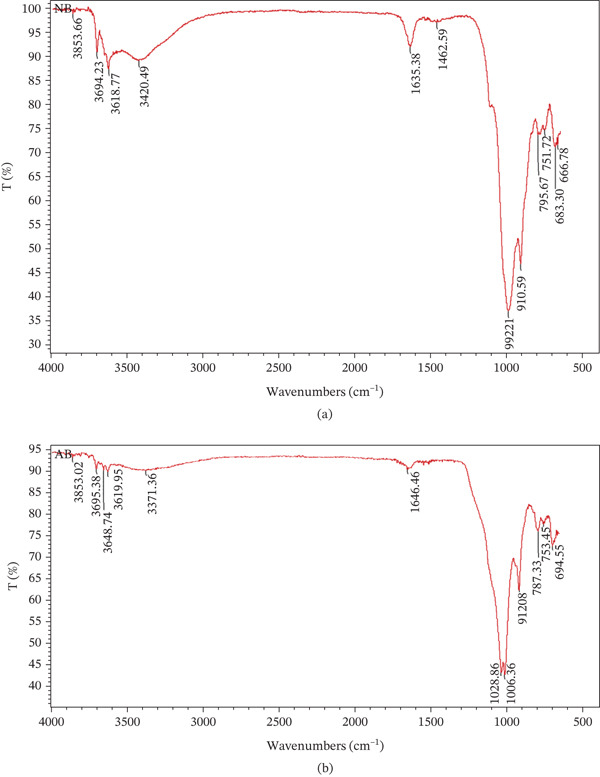
Absorption bands: (a) NLBn and (b) ALBn.

The bonding characteristics of NLBn and ALBn were investigated using FT‐IR spectroscopy over the wave number range of 500–4000 cm^−1^, with a Thermo Scientific Nicolet iS10 FT‐IR Spectrometer, as shown in Figure 4. The figure illustrates the typical absorption bands across all spectra. The [(CH_2_) _n_] cluster in NLBn, primarily observed between 683.30 and 759.67 cm^−1^, was attributed to these absorption peaks. However, these bands shifted slightly in ALBn, with peaks appearing around 694.55 and 787.33 cm^−1^. Additionally, the (Al–O–Si) cluster in ALBn displayed a slight shift, with its band located at 912.08 cm^−1^. In comparison, the same cluster in NLBn appeared between 910.59 and 992.21 cm^−1^, following an activation treatment. This shift in NLBn is likely due to a natural alteration in its chemical structure, which involves the transformation of the tetrahedral layer when Ca^+2^ enters to form an amorphous oxide through acid activation. This process is reflected by an increase in peak intensity, suggesting that more surface adsorption sites may have been created. The (–OH) cluster in NLBn was identified at 1635.38 cm^−1^, corresponding to water deformation, whereas in ALBn, it was attributed to dehydroxylation at 3619.95 cm^−1^, which typically results from the acid activation of the clay. The stretching bands for (SiO4 and SiO2) and (OH–AL–OH) appeared in the same range (910.59–992.21 cm^−1^) for both types of clay. Furthermore, the O–Si band in ALBn appeared at 1028.86 cm^−1^, whereas for NLBn, this band was absent before activation, indicating that the band formation in ALBn was a result of the activation process [14].

#### 3.1.3. SEM Images of the Prepared Adsorbents

**Figure 5:**
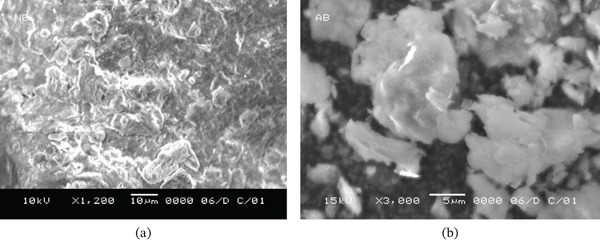
SEM images of the morphologies of (a) NLBn and (b) ALBn.

This study was carried out at the IRC in Tripoli, Libya, using the JEOL‐JSM‐5610LV. SEM images, as shown in Figure 5a,b, illustrate a marked contrast between the natural clay surfaces, which are predominantly smooth and uniform, and the surfaces subjected to activated functionalization. The images offer clear visual evidence of the newly formed regions resulting from the applied treatment. The treated samples reveal the presence of several newly created voids, suggesting that the surface treatment likely facilitated the removal of certain acid‐soluble salts, further altering the material structure.

### 3.2. Effect of Duration of Contact

**Figure 6:**
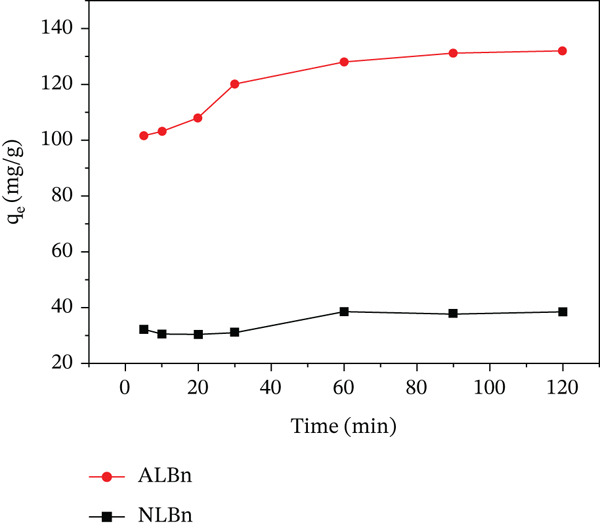
Effect of contact time on CR adsorption capacity of NLBn and ALBn at initial concentration = 50 ppm, pH = 6.9, temperature = 25^°^C ± 2^°^C, and dosage = 0.13 g/L.

The adsorption investigation was performed over various time periods ranging from 5 to 120 min to identify the equilibrium contact time for the adsorption of CR on NLBn and ALBn adsorbents. Figure 6 shows the adsorption of CR on NLBn and ALBn; it is clear that adsorption increases as contact time increases up to 120 min. NLBn and ALBn had maximum adsorption capacities of 38 and 132 mg/g for CR, respectively, suggesting that adsorption begins quickly for ALBn and slowly for NLBn due to macro‐ and micropores of the adsorbent. The reduced adsorption rate is caused by a reduction in the number of empty sites on the adsorbent [21, 22]. Later, due to the highly negatively charged surface of the clay, which favors cationic adsorption rather than anionic dyes under the same conditions, NLBn was found to have a lower adsorption capacity than ALBn [23].

### 3.3. Effect of pH

**Figure 7:**
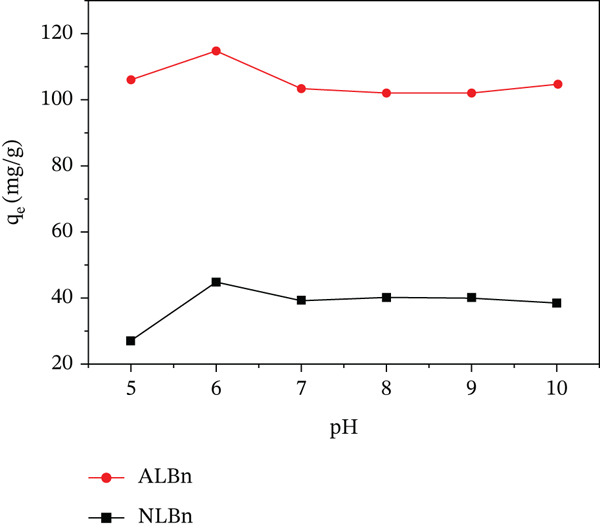
Effect of pH on the adsorption capacity (mg/g) of CR by NLBn and ALBn at initial concentration = 50 ppm, temperature = 25 ±  ^°^C, contact time 60 min, and dosage = 0.13 g/L.

**Figure 8:**
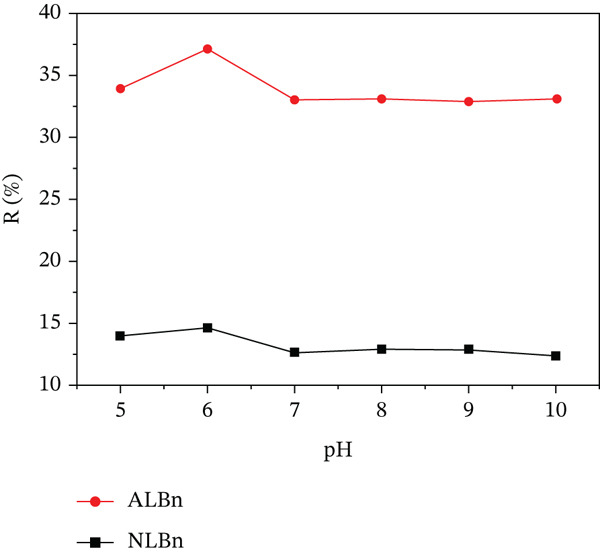
Effect of pH on CR removal by NLBn and ALBn at initial concentration = 50 ppm, temperature = 25^°^C ± 2^°^C, contact time = 60 min, and dosage = 0.13 g/L.

The effect of pH on the adsorption of CR onto NLBn and acid‐activated Libyan bentonite was investigated across a pH range of 5.0–10.0, as illustrated in Figures 7 and 8. The adsorption capacity increased from 27.2 to 44.8 mg/g for NLBn and from 105.6 to 114.4 mg/g for ALBn when the pH of the solution reached 6. After that, changes in pH had little impact on the adsorption for both types of bentonites in terms of the amount of dye adsorbed (*q*_*e*_) and the percentage removed (*R* *%*). This behavior may be attributed to the interaction between the positively charged surface of the adsorbent and the anionic nature of CR dye [23]. In the pH range of 7–10, the surface charge and electrostatic interactions appear to stabilize, resulting in negligible variation in adsorption performance. The observed shift in the solution′s red coloration is linked to resonance effects within the dye′s charged molecular structure. Additionally, although bentonite is inherently basic, exposure to inorganic acids during activation imparts a negative surface charge, further enhancing its adsorption potential.

### 3.4. Effect of Adsorbent Dosage

**Figure 9:**
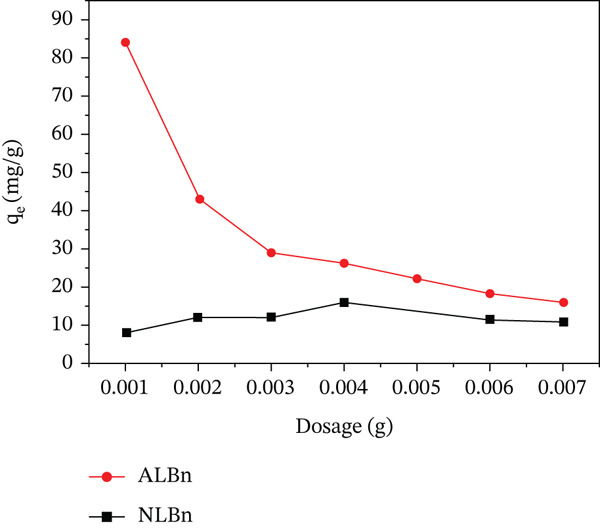
Effect of dosage on CR adsorption capacity by NLBn and ALBn at initial concentration = 50 ppm, contact time = 60 min, temperature = 25^°^C ± 2^°^C, and pH = 6.9.

Figure 9 shows the tested adsorbent doses of 0.001, 0.002, 0.003, 0.004, 0.005, 0.006, and 0.007 g/L. By gradually increasing the dosage of NLBn and ALBn, the results show that ALBn exhibits a significantly higher adsorption capacity, reaching approximately 85 mg/g at 0.001 g/L dosage. However, as the dosage increases, it, sharply declines to around 30 mg/g at 0.002 g/L and continues decreasing gradually to 10 mg/g at 0.007 g/L likely due to site saturation. In contrast, NLBn shows a lower and more stable adsorption capacity, ranging from 5 to 10 mg/g across the tested dosages. This can be explained by depilation of microspores from the surface. Both clays have the same properties (pores are completely filled with dye solution), but other bentonite treatments, including acid and thermal activation, may be more successful in absorbing CR [24].

### 3.5. Effect of Initial Dye Concentration

**Figure 10:**
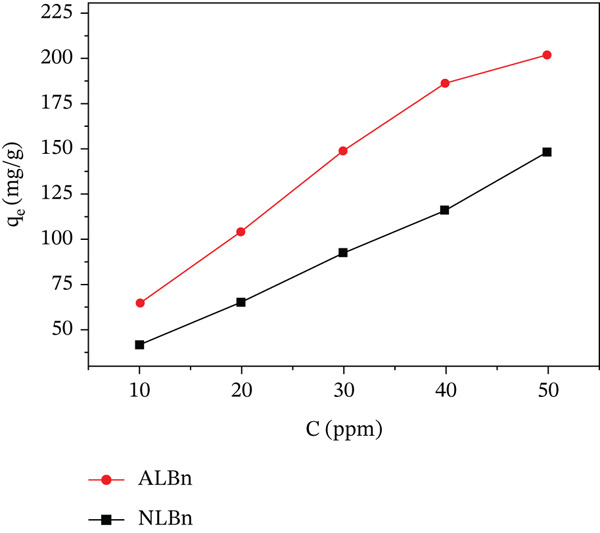
Effect of initial concentration on capacity of CR by NLBn and ALBn at 25^°^C ± 2^°^C, pH = 6.9, and contact time = 60 min.

Figure 10 illustrates the dependence of CR dye adsorption on the initial dye concentration, ranging from 10 to 60 mg/L. The characteristic shape of the isotherms for both the natural (NLBn) and activated (ALBn) bentonite samples is consistent with the L‐type (Langmuirian) isotherm classification proposed by Giles et al. [25]. This result suggests a significant adsorbate attraction for the surface sites at low concentrations, followed by the progressive saturation of a monolayer at higher levels, which is similar to a previous investigation on CR adsorption onto modified bentonite [26].

The consistent increase in the equilibrium adsorption capacity *q*_*e*_ with rising initial concentration confirms that a higher concentration gradient facilitates mass transfer, thereby enhancing the diffusion of CR molecules onto the adsorbent surface. Throughout the tested range, ALBn exhibited significantly higher *q*_*e*_ values than NLBn.

This result indicated that the activation process successfully optimized the surface characteristics, specifically increasing porosity and greatly increasing the total number of active adsorption sites [23]. This finding of notable adsorbate attraction for surface sites at low concentrations, followed by progressive monolayer saturation at higher levels, clearly demonstrates that chemical activation extensively increases bentonite′s intrinsic adsorption, making ALBn a more competitive and effective adsorbent for the removal of organic dyes from aqueous media.

**Figure 11:**
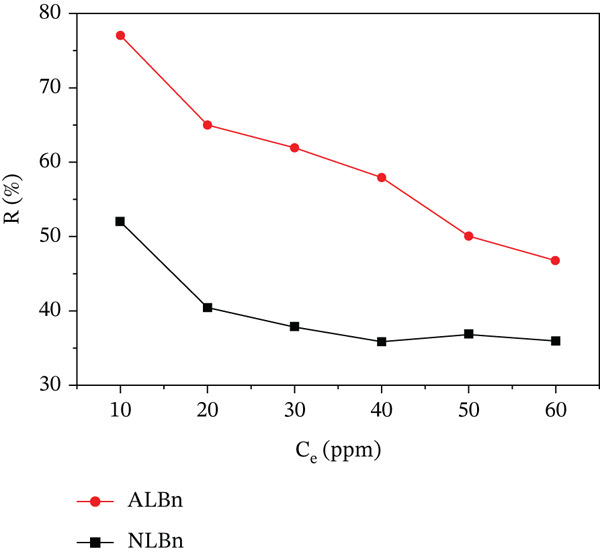
Effect of initial concentration on CR removal by NLBn and ALBn at 25^°^C ± 2^°^C, pH = 6.9, contact time = 60 min, and dosage = 0.13 g/L.

Figure 11 illustrates the decline in removal efficiency (*R* *%*) with increasing initial concentrations of CR. This is attributed to the limited availability of active sites relative to the higher quantity of adsorbate molecules. While both samples followed similar trends, ALBn consistently displayed a higher adsorption capacity under identical conditions. Specifically, the removal efficiency for the ALBn adsorbent decreased from a maximum of 77% to 39%, whereas that for NLBn dropped from 52% to 21%, emphasizing the superior performance gained through activation.

### 3.6. Effect of Temperature

**Figure 12:**
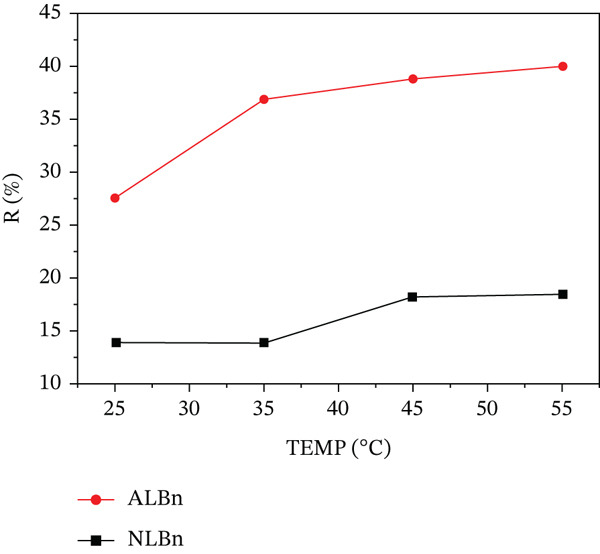
Effect of temperature on CR removal by NLBn and ALBn at initial concentration = 50 ppm, pH = 6, contact time = 60 min, and dosage = 0.13 g/L.

As shown in Figure 12, increasing the temperature from 25°C to 55°C enhanced the adsorption of CR onto both NLBn and acid‐activated Libyan bentonite. Positive Gibbs free energy values indicated nonspontaneous adsorption, with ALBn showing a stronger affinity for CR and higher disorder at the solid–solution interface. These results align with previous studies, highlighting ALBn′s superior adsorption properties [9].

### 3.7. Adsorption Kinetics

The adsorption kinetics of CR were evaluated using the first‐order Lagergren model (Equation 3) and the pseudo‐second‐order model (Equation 4).

(3) lnqt−qe=lnqe−k1t2.303

(4) tqt=1K2qe2+tqe

**Figure 13:**
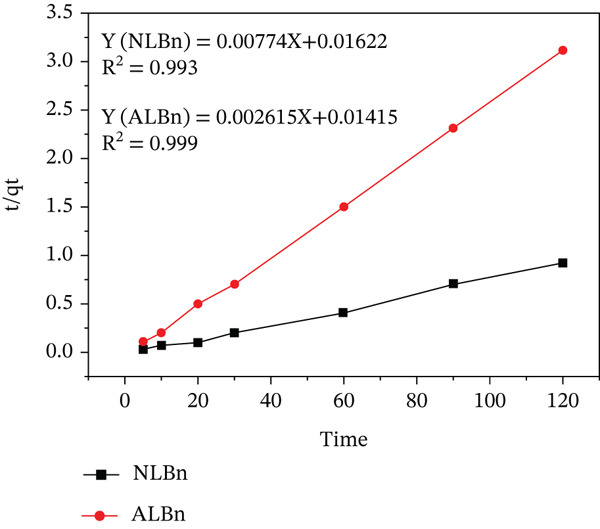
Pseudo‐second‐order adsorption of CR adsorbed on NLBn and ALBn at initial concentration = 50 ppm, temperature = 25^°^C ± 2^°^C, pH = 6.99, and dosage = 0.13 g/L.

The first‐order model describes adsorption rate regularity, with the rate constant (*k*_1_) determined from the slope of a plot of *t* versus ln (*q*_*e*_ − *q*_*t*_). The pseudo‐second‐order model, assuming chemisorption, best fits the experimental data. The second‐order rate constant (*K*_2_) was determined by plotting *t*/*q*_*t*_ versus *t*. Results showed that the pseudo‐second‐order model provided the best fit for both NLBn and ALBn, with *R* ^2^ values near 1 as shown in Figure 13 and Table 3. This indicates excellent agreement between theoretical and experimental data, confirming the model′s suitability for describing CR adsorption on clay [24, 27].

**Table 3:** Kinetic parameter for the adsorption of CR onto NLBn–ALBn clays.

**Pseudo-first-order kinetic model**			
**Sample**	**q_e_ (mg/g)**	**K_1_ (g mg ^−1^ min ^−1^)**	${R_{1}}^{2}$
*NLBn*	128	0.729	0.803
*ALBn*	38.400	0.227	0.757
**Pseudo-second-order kinetic model**			
**Sample**	**q_e_ (mg/g)**	**K_2_ (g mg^−1^ min^−1^)**	${R_{2}}^{2}$
*NLBn*	129.200	0.0037	0.993
*ALBn*	377.400	0.0005	0.999

### 3.8. Adsorption Isotherms

This study explored the adsorption of CR onto NLBn and ALBn using Langmuir, Freundlich, and Temkin adsorption isotherms.

(5) Ceqe=1bqm+Ceqm

(6) nqe=lnkf+1nlnCe

(7) qe=kTln Ce+BT

**Figure 14:**
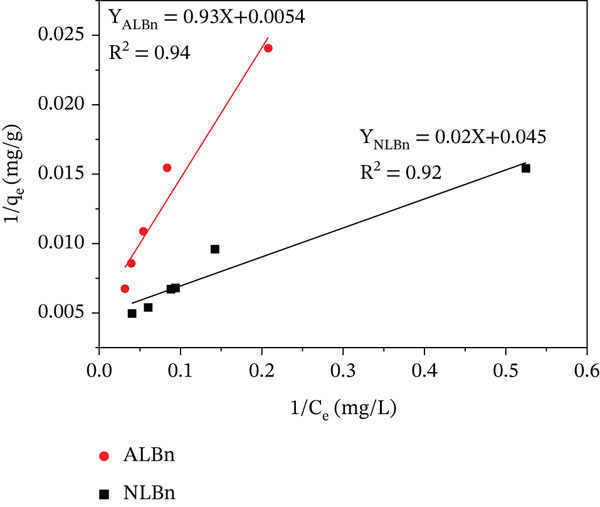
Langmuir adsorption isotherm for CR on NLBn and ALBn at pH = 6.9, temperature = 25^°^C ± 2^°^C, and dosage = 0.13 g/L.

**Figure 15:**
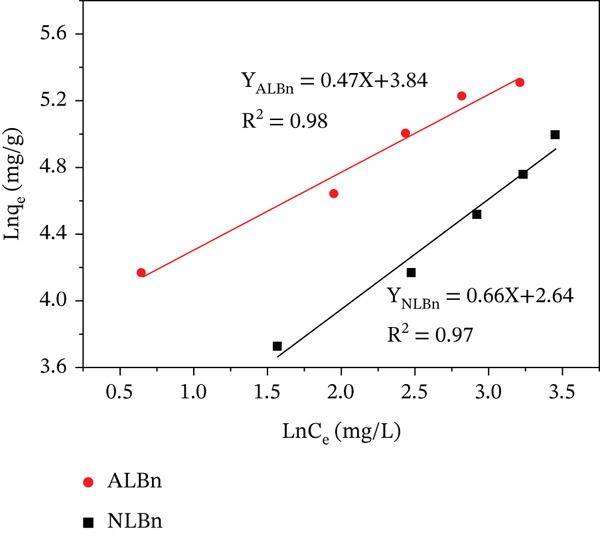
Freundlich adsorption isotherm for CR on NLBn and ALBn at pH = 6.9, temperature = 25^°^C ± 2^°^C, and dosage = 0.13 g/L.

**Figure 16:**
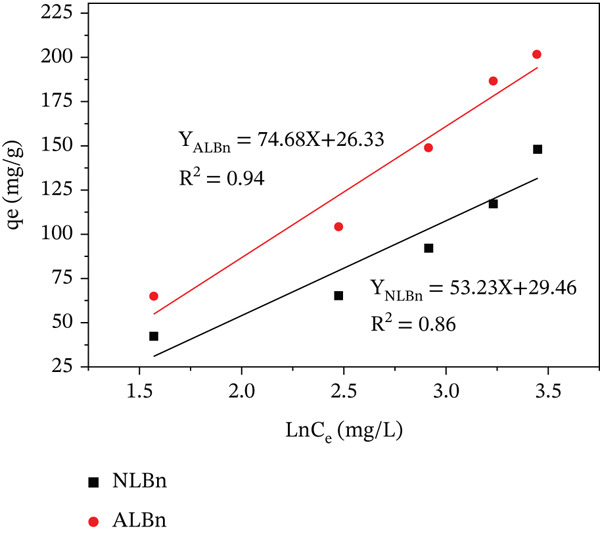
Temkin adsorption isotherm for CR on NLBn and ALBn at pH = 6.9, temperature = 25^°^C ± 2^°^C, and dosage = 0.13 g/L.

The adsorption processes and interactions between the adsorbents and CR molecules were better understood by fitting the experimental data to these models (Equations (5)–(7)). The Temkin model includes parameters for adsorption heat (*K*_*T*_ and *B*_*T*_), the Freundlich model correlates adsorption intensity and capacity (*K*_*f*_ and 1/*n*), and the Langmuir model describes the highest and equilibrium adsorption capacities (*q*_*e*_ and *q*_max_). Applying these models to the experimental data provided the parameters illustrated in Figures 14, 15, and 16.

It is evident that the Freundlich model provided better representations of the adsorption data for NLBn and ALBn, respectively. The evidence was supported by models with higher coefficient values (*R* ^2^) than those of competing models. The calculated values of RL for adsorption of CR fall between (0) and (1) (Table 4); Therefore, the adsorption process of dye onto NLBn and ALBn was favorable. Similar to what is observed when natural Iraqi bentonite where obeys the Freundlich model (*R* ^2^ = 0.9978). The mathematical superiority of the Freundlich model, despite the visually observed L‐type curve (monolayer tendency), suggests that the adsorption occurs primarily on a heterogeneous surface with diverse adsorption sites and varying energies [28]. This observed heterogeneity is likely a direct consequence of the chemical activation process, which involves acid leaching and structural reorganization, thus generating a broader spectrum of active sites on the bentonite surface.

**Table 4:** Langmuir, Freundlich, and Temkin parameters of NLBn and ALBn.

Model	Parameter	NLBn	ALBn
Langmuir	*q*_max_ (mg/g)	201.6	148
	*K*_*L*_ (L/mg)	2.49 × 10^−5^	0.33
	*R*_*L*_	0.99	0.05
	*R* ^2^	0.92	0.94

Freundlich	*K*_*F*_ (mg/g)	3.84	3.41
	*n*	1.50	2.15
	*R* ^2^	0.98	0.97

Temkin	*K*_*T*__(mg/g)_	0.38	1.39
	*B*_*T*_	53.74	55.50
	*R* ^2^	0.86	0.94

### 3.9. Thermodynamic Studies

Thermodynamic parameters were added to enhance the explanation of the impact of temperature on the adsorption of CR onto NLBn and ALBn. The following equations (Equations (8)–(10)) were used to study the thermodynamic parameters: change in free energy (*Δ* *G*), change in enthalpy (*Δ* *H*), and change in entropy (*Δ* *S*) [20, 26, 29, 30].

(8) ΔG=−RTlnKD

(9) lnKD=ΔSR−ΔHRT

(10) lnKD=qeCe

**Figure 17:**
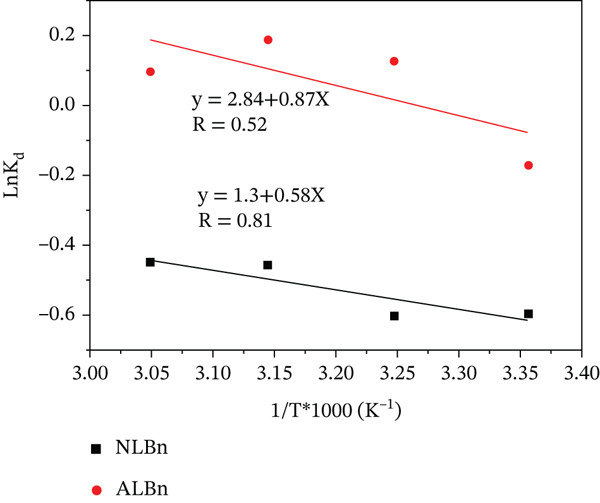
Estimation of thermodynamic parameters for CR by NLBn and ALBn.

The partition coefficient (*K*_*D*_), ideal gas constant (*R* = 8.314 J mol^−1^ K^−1^), and absolute temperature (*T* in Kelvin) were used to analyze the adsorption of CR on NLBn and ALBn. A plot of ln*K*_*D*_ versus 1/*T* (Figure 17) was used to calculate the values of *Δ* *H* and *Δ* *S*, with *Δ* *G* determined using Equation (8). The results, listed in Table 5, show that CR adsorption is endothermic and spontaneous, with positive *Δ*H and negative *ΔG* values. The positive *ΔS* value suggests that CR is randomly adsorbed on the surface, reflecting the energy changes involved in the adsorption process. These findings indicate that CR adsorption on bentonite occurs spontaneously at low concentrations [31–34].

**Table 5:** Thermodynamic parameters for adsorption of CR at different temperatures.

Adsorbent	*Δ* *G*(J·mol^−1^)				*Δ* *S* ^*o*^(J·mol−^1^·K^−1)^	*Δ* *Η* ^*o*^(J·mol^−1^·K)
	25°C	35°C	45°C	55°C		
NLBn	−3224.2	−3332.6	−3440.9	−3549.3	10.8	4.70
ALBn	−7016.9	−7252.6	−7488.3	−7724.1	23.6	7.23

### 3.10. Comprehensive Comparison Between Libyan Bentonite and Other World Clay

Adsorption method research is still in great demand because of its applicability and minimal requirements for laboratory work. The treatment of pollutants [35, 36] and medicine [28, 29] are two important uses of this technology, as mentioned in the introduction. To create inexpensive, easily accessible, nontoxic adsorbents for the removal of dyes from aqueous solutions, a range of materials are used as adsorbents for different hues in a number of industries [37–39]. Adsorption amounts [4] can be explained by the relative energies of the adsorbent–adsorbate, adsorbate–solvent, and adsorbate–adsorbate interactions. The adsorption of dye on the bentonite surface in our investigation may benefit from these findings. Tables 6 and 7 present a summary of some comprehensive comparisons between Libyan bentonite and other world bentonite clays for the adsorption of Congo red from aqueous solution.

**Table 6:** Comprehensive comparison between natural Libyan bentonite, activated Libyan, and some world bentonite clay for the adsorption of CR in aqueous solution.

Clay	Effect of pH	Effect of equilibrium time (min)	Adsorption capacity (mg·g^−1^)	Isotherm model OBYES at 25^o^C	Kinetic model	Ref.
LNBn	*p* *H* = 6*R* = 14.7*%*	90–120	38	Freundlich	Pseudo‐second order (*R* = 0.993)	This study
LABn	*p* *H* = 6*R* = 36.8	90–120	132	Freundlich	Pseudo‐second order (*R* = 0.999)	This study
Agadir clay	*p* *H* = 2*R* = 98.97*%*	60–120	91	Langmuir	Pseudo‐first order (*R* = 0.926)	[9]
Iraqi bentonite	*p* *H* = 4.8*R* = 87.9	70–60	23	Freundlich	Pseudo‐second order (*R* = 0.997)	[40]
Egyptian bentonite	5–10	90–120	34	Langmuir	Pseudo‐second order (*R* = 0.997)	[41]

**Table 7:** Thermodynamic parameter of The CR adsorption to world bentonite clay.

Clays	Dye	*Δ* *G*(kJ·mol^−1^)				*Δ* *H* ^°^(kJ·mol^−1^)	*Δ* *S* ^°^(J mol^−1^ K^−1^)	Ref
		25°C	35°C	45°C	55°C			
LNBn	MB	−8.433	−8.716	−8.999	−9.282	0.730	28.30	[12]
	CR	−3.224	−3.333	−3.441	−3.549	4.700	10.810	This study
LABn	MB	−9.195	−9.503	−9.812	−10.121	0.865	30.85	[12]
	CR	−7.017	−7.253	7.488	−7.724	23.600	7.23	This study
AC (Agadir clay)	MB	−1.316	−1.822	−2.199	−2.357	23.650	92.66	[7]
	CR	−0.455	0.296	−0.093	0.052	−9.082	−27.56	[7]
Iraqi bentonite	MB	−14.783				−32.040	−5.17	[40]
	CR	−2.003	−2.506	−3.105	−4.052	19.126	−70.90	[41]
Egyptian bentonite	CR	−21.104	−22.191	−23.352	/	12.380	0.134	[42]

## 4. Conclusion

This study examined the adsorption behavior of CR on natural (NLBn) and acidified bentonites (ABN) from the Umm Al‐Razzam area. The results showed that the removal efficiency improved with increasing equilibrium adsorption constant, especially in the relevant dose range. ALBn removed 5.4% of the sample and NLBn 4.9%, showing that ALBn forms a monolayer and NLBn adsorbs in several layers in an anionic medium. The ALBN had a higher enthalpy, which made the adsorption process more spontaneous and beneficial. Positive values of *Δ* *S* ^°^ indicated greater disorder at the solid–solution interface. Freundlich isotherms were consistent with NLBn, whereas Langmuir was the best characterizer for ALBn. The adsorption process followed the paradigm of a pseudo‐second‐order model. Both bentonites showed increased adsorption of the dye at higher pH levels, although the pH change from 7 to 10 had only a minimal effect.

## Author Contributions

This research was conducted through the collaborative efforts of the following contributors:

**Salha M. Aghila:** designed the study, conducted experiments, analyzed adsorption kinetics manuscript preparation. **Hana A. Jamhour**: writing the paper, assisted in sample preparation, data collection, and optimization of adsorption conditions. **Abdulhakim A. Jangher**: research supervisor and manuscript editing. **Basher M. Mahara**: manuscript editing. **Ibrahim M. Hamammu:** provided expertise statistical analysis and manuscript editing. **Daefalla M. Tawati**: contributed to manuscript preparation and manuscript editing.

## Funding

No funding was received for this manuscript.

## Conflicts of Interest

The authors declare no conflicts of interest.

## Data Availability

The data that support the findings of this study are available on request from the corresponding author. The data are not publicly available due to privacy or ethical restrictions.
